# A BAHD acyltransferase contributes to the biosynthesis of both ethyl benzoate and methyl benzoate in the flowers of *Lilium* oriental hybrid ‘Siberia’

**DOI:** 10.3389/fpls.2023.1275960

**Published:** 2023-09-29

**Authors:** Yuechong Yue, Lan Wang, Manyi Li, Fang Liu, Junle Yin, Lijun Huang, Bin Zhou, Xinyue Li, Yunyi Yu, Feng Chen, Rangcai Yu, Yanping Fan

**Affiliations:** ^1^ The Research Center for Ornamental Plants, College of Forestry and Landscape Architecture, South China Agricultural University, Guangzhou, China; ^2^ Guangdong Key Laboratory for Innovative Development and Utilization of Forest Plant Germplasm, South China Agricultural University, Guangzhou, China; ^3^ College of Life Sciences, South China Agricultural University, Guangzhou, China; ^4^ Department of Plant Sciences, University of Tennessee, Knoxville, TN, United States

**Keywords:** lily, floral scent, BAHD acyltransferase, methyl benzoate, ethyl benzoate, biosynthesis

## Abstract

Lily is a popular flower worldwide due to its elegant appearance and pleasant fragrance. Floral volatiles of lily are predominated by monoterpenes and benzenoids. While a number of genes for monoterpene biosynthesis have been characterized, the molecular mechanism underlying floral benzenoid formation in lily remains unclear. Here, we report on the identification and characterization of a novel BAHD acyltransferase gene that contributes to the biosynthesis of two related floral scent benzoate esters, ethyl benzoate and methyl benzoate, in the scented *Lilium* oriental hybrid ‘Siberia’. The emission of both methyl benzoate and ethyl benzoate in *L.* ‘Siberia’ was found to be tepal-specific, floral development-regulated and rhythmic. Through transcriptome profiling and bioinformatic analysis, a BAHD acyltransferase gene designated *LoAAT1* was identified as the top candidate gene for the production of ethyl benzoate. *In vitro* enzyme assays and substrate feeding assays provide substantial evidence that LoAAT1 is responsible for the biosynthesis of ethyl benzoate. It was interesting to note that in *in vitro* enzyme assay, LoAAT1 can also catalyze the formation of methyl benzoate, which is typically formed by the action of benzoic acid methyltransferase (BAMT). The lack of an expressed putative BAMT gene in the flower transcriptome of *L.* ‘Siberia’, together with biochemical and expression evidence, led us to conclude that LoAAT1 is also responsible for, or at least contributes to, the biosynthesis of the floral scent compound methyl benzoate. This is the first report that a member of the plant BAHD acyltransferase family contributes to the production of both ethyl benzoate and methyl benzoate, presenting a new mechanism for the biosynthesis of benzoate esters.

## Introduction

Lily is one of the most popular flowers worldwide with tremendous ornamental and economic values (Bakhshaie et al., 2016). More than 10,000 lily cultivars have been bred, and they present a wide diversity of flower color, shape, size, and scent (Younis et al., 2014). Based on the composition of major scent components in 41 cultivars, the lily floral scent was classified into six aroma types: faint-scented, cool, fruity, musky, fruity-honey, and lily, ranging from weakly scented to strongly fragrant (Du et al., 2019). More than sixty floral volatiles have been identified in scented lily cultivars with monoterpenes and benzenoids being most abundant (Kong et al., 2012; Johnson et al., 2016; Kong et al., 2017). The lily monoterpene biosynthetic pathway has been well investigated using gas chromatography-mass spectrometry (GC-MS) and RNA-seq methods (Du et al., 2017; Hu et al., 2017; Shi et al., 2018), and several key genes involved in floral monoterpene biosynthesis have been characterized using *in vitro* and/or *in planta* methods (Zhang et al., 2018; Abbas et al., 2019; Zhang et al., 2020). In contrast, little is known about the molecular mechanism of the biosynthesis of benzenoids in lily flowers.

Benzenoid biosynthesis in plants originates from phenylalanine, which is first deaminated to form cinnamic acid by phenylalanine ammonia-lyase (PAL) (Skaliter et al., 2022). Subsequent conversion of cinnamic acid to the precursor for volatile benzenoids, benzoyl-CoA/benzoic acid is achieved through the β-oxidation pathway and/or the non-β-oxidation pathway (Widhalm and Dudareva, 2015). Thereafter, the formation of benzenoid methyl esters is known to be catalyzed by methyltransferase of the SABATH family, which takes its name from the first three biochemically characterized enzymes, salicylic acid methyltransferase (SAMT), BAMT, and theobromine synthase (D'Auria et al., 2003; Muhlemann et al., 2014). The SABATH family is a distinct class of methyltransferases in plants, members of which catalyze the generation of a number of volatile methyl esters, including methyl benzoate, methyl salicylate, methyl anthranilate and methyl cinnamate (D'Auria et al., 2003; Effmert et al., 2005; Kollner et al., 2010; Zhang et al., 2019). Methyl benzoate, which has a fruity odor, is a common ingredient of floral volatiles and has been identified in over eighty species (Knudsen et al., 2006). Methyl benzoate in plants is normally generated as a result of the methylation of benzoic acid by the action of BAMT of the SABATH family (D'Auria et al., 2003; Effmert et al., 2005). To date, the SABATH methyltransferases responsible for floral methyl benzoate biosynthesis have been characterized in several species, including snapdragon (Dudareva et al., 2000), petunia (Negre et al., 2003), *Nicotiana* (Hippauf et al., 2010), and *Hedychium coronarium* (Yue et al., 2021).

Biosynthesis of benzenoid acyl esters is catalyzed by the BAHD acyltransferases (Muhlemann et al., 2014). As a large group of acyl-CoA-dependent enzymes, the BAHD family is involved in the acylation of a diverse groups of alcohols (Wang et al., 2022). The BAHD acyltransferase family is named based on the first four biochemically characterized enzymes of this family, benzyl alcohol *O*-acetyltransferase (BEAT), anthocyanin *O*-hydroxycinnamoyltransferase (AHCT), anthranilate *N*-hydroxycinnamoyl/benzoyltransferase (HCBT) and deacetylvindoline 4-*O*-acetyltransferase (DAT) (St-Pierre and De Luca, 2000; D'Auria, 2006). Phylogenetic analysis of plant BAHD acyltransferases revealed eight clades for the family, and the alcohol acyltransferases (AATs) responsible for volatile ester biosynthesis fell into clade IIIa and Va (D'Auria, 2006; Tuominen et al., 2011). The AATs within clade IIIa mainly utilize acetyl-CoA as the acyl donor, while the AATs that are capable of generating benzenoid ester cluster within clade Va (D'Auria, 2006; Tuominen et al., 2011). For example, *Clarkia breweri* BEBT, in clade Va, catalyzes the formation of floral benzylbenzoate using benzyl alcohol and the acyl donor benzoyl-CoA (D'Auria et al., 2002). Furthermore, it is worth noting that some AATs can accept a diverse range of alcohol substrates and produce more than one volatile ester *in planta*, such as apricot PaAAT1, which is responsible for the production of (*E*)-2-hexenyl acetate and (*Z*)-3-hexenyl acetate during fruit ripening (Zhou et al., 2021). Ethyl benzoate with a somewhat fruity odor is present in the floral scents of plants among more than twenty different families (Knudsen et al., 2006) and is one of the dominant floral volatiles in some scented lily cultivars (Kong et al., 2012). However, its biosynthesis in flowers is scarcely mentioned. Only in ripe kiwifruit, the volatile ester ethyl benzoate was demonstrated to be formed through the benzoylation of ethanol by alcohol acyltransferase AT16 (Gunther et al., 2011). Thus, it is necessary to elucidate the molecular mechanism for the formation of ethyl benzoate in floral scent profiles.

It is interestingly to note that besides SABATH methyltransferases, certain BAHD acyltransferases have been reported to participate in the biosynthesis of methyl esters. For example, a grape BAHD acyltransferase AMAT is responsible for the production of methyl anthranilate using methanol and anthraniloyl-CoA as substrates (Wang and De Luca, 2005). In addition, overexpressing apple *MdAAT2* in transgenic tobacco leaves led to significant increase of methyl benzoate concentration and new formation of other methyl esters (Li et al., 2008). It remains to be determined whether the contribution to methyl ester biosynthesis by BAHD acyltransferase is wide spread in plants.

Among the diverse lily cultivars, *Lilium* ‘Siberia’, a world-famous artificially cultivated oriental hybrid, is known for its large, white and fragrant flowers. Both methyl benzoate and ethyl benzoate are important constituents of its floral scent (Kong et al., 2012; Shi et al., 2018). Therefore, *Lilium* ‘Siberia’ is selected as a model plant to investigate the molecular mechanisms underlying the biosynthesis of floral benzoate esters. In this study, the emission patterns of benzoate esters in *L.* ‘Siberia’ were investigated, and candidate genes for floral benzenoid biosynthesis were identified using RNA-seq. Furthermore, the *LoAAT1* gene critical for floral benzoate esters formation was functionally characterized. Finally, the mechanism underlying the variation in the amount of floral benzoate esters released among different cultivars was investigated and discussed.

## Results

### Tepal-specific, floral development-regulated and rhythmic emission of methyl benzoate and ethyl benzoate

**Figure 1:**
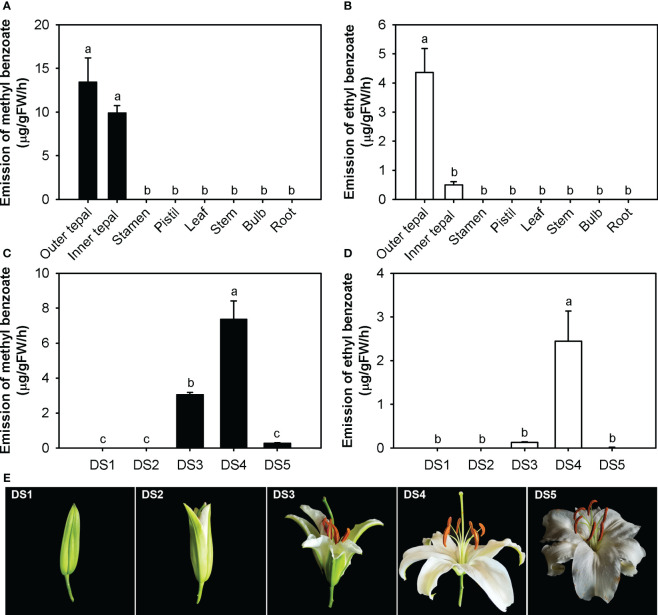
Tepal-specific and floral development-regulated emission of methyl benzoate and ethyl benzoate in *L.* ‘Siberia’. **(A, B)** Emission of methyl benzoate **(A)** and ethyl benzoate **(B)** in different tissues of *L.* ‘Siberia’. **(C, D)** Changes in floral methyl benzoate **(C)** and ethyl benzoate **(D)** emissions during flower development. **(E)** Representative images of flowers in different floral developmental stages. Error bars indicate the standard deviation of three biological replicates. Different lowercase letters labelled on bars indicate statistically significant differences at the level of *P* < 0.05.

To investigate the methyl benzoate and ethyl benzoate released in *L.* ‘Siberia’, their spatiotemporal and rhythmic emission patterns were analyzed using solid phase microextraction (SPME) collection and GC-MS analysis. Of the eight tissues examined, only the outer tepals and inner tepals released methyl benzoate and ethyl benzoate (**Figures 1A, B**), indicating a tepal-specific emission pattern. No benzoate esters were detected in the stamen, pistil or nonfloral organs, including the leaf, stem, bulb and root (**Figures 1A, B**). During flower development, methyl benzoate and ethyl benzoate were hardly detectable in the DS1 and DS2 stages (**Figure 1C-E**). The emission of methyl benzoate increased sharply in the process of flower opening, reached a maximum at the full-opening stage (DS4) and then declined to a low level in the senescence stage (DS5, **Figures 1C, E**). For ethyl benzoate, the highest emission rate was also observed at the full-opening stage (DS4). However, only a slight amount of ethyl benzoate was emitted in the DS3 and DS5 stages (**Figures 1D, E**).

**Figure 2:**
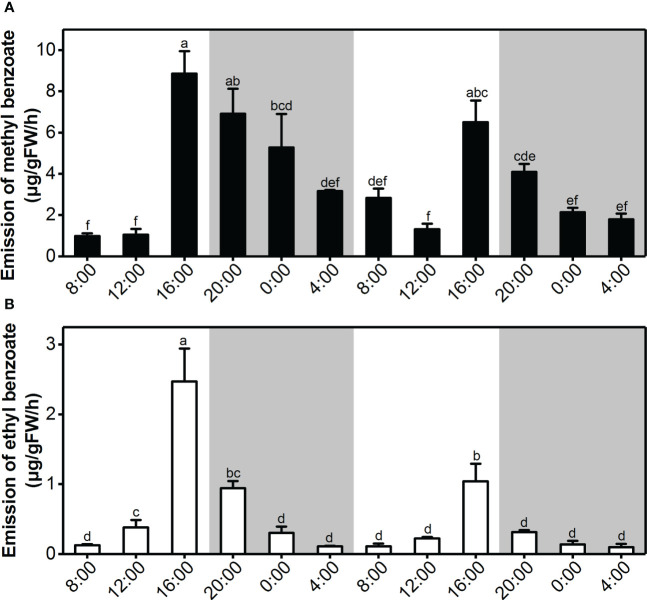
Rhythmic emission of methyl benzoate and ethyl benzoate in *L.* ‘Siberia’ flowers. **(A)** Changes in floral volatile methyl benzoate **(A)** and ethyl benzoate **(B)** during two consecutive light/dark (12-h/12-h) cycles on the day after full-opening. White and grey areas correspond to light and dark, respectively. Error bars indicate standard deviation of three biological replicates. Different lowercase letters labeled on bars indicate the statistically significant differences at the level of *P* < 0.05.

Previous observations showed that *L.* ‘Siberia’ flowers are more fragrant at dusk than at dawn (Shi et al., 2018; Abbas et al., 2019). Thus, we performed a detailed time-course analysis of benzoate ester emission under two daily light/dark cycles. The methyl benzoate emission was maintained at a relatively low level from 4:00 to 12:00, peaked at 16:00 and decreased gradually thereafter (**Figure 2A**), displaying rhythmic variation. Emission of ethyl benzoate changed rhythmically during two daily light/dark cycles, with a maximum emission at 16:00 and a minimum emission at 4:00 (**Figure 2B**). Thus, the emission of methyl benzoate and ethyl benzoate was tepal-specific, floral development-regulated and rhythmic.

### Transcriptome profiling identified candidate genes for the floral benzenoid pathway

**Figure 3:**
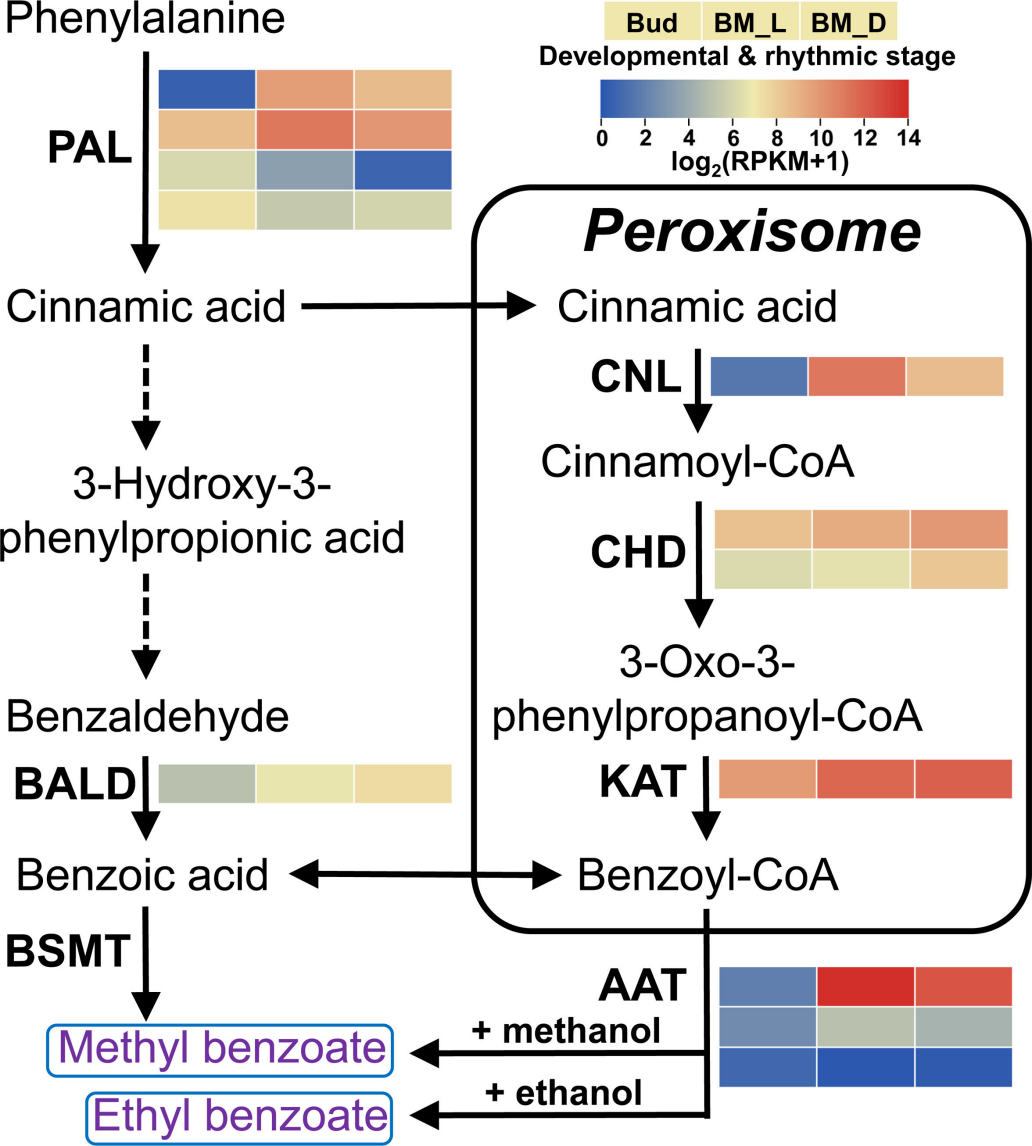
Expression profiles of genes encoding enzymes possibly involved in the biosynthesis of methyl benzoate and ethyl benzoate in *L.* ‘Siberia’. The enzyme’s abbreviated name for each catalytic step is shown in bold. Broken arrows represent proposed catalytic steps not yet described in plants. Gene expression levels (RPKM+1) with log_2_ transformation are represented by color gradation. The three color blocks in the row signify the tepal samples in the bud stage, the blooming stage at 16:00 (BM_L) and the blooming stage at 4:00 (BM_D). The color blocks in the column represent different homologue genes that are termed in Arabic numerical order from top to bottom. AAT, alcohol acyltransferase; BALD, benzaldehyde dehydrogenase; BSMT, benzoic acid/salicylic acid carboxyl methyltransferase; CHD, cinnamoyl-CoA hydratase-dehydrogenase; CNL, cinnamoyl-CoA ligase; PAL, phenylalanine ammonialyase; KAT, 3-ketoacyl CoA thiolase.

To identify the genes involved in the biosynthesis of floral benzenoids, we sequenced and assembled the *L.* ‘Siberia’ transcriptome, which contained 29,837 unigenes with an N50 value of 1,284 bp (**Supplementary Tables 1, 2**). Using a local BLAST search against the current assembled lily transcriptome, nine complete genes possibly involved in benzenoid biosynthesis were identified, including four *PAL* genes, four β-oxidative pathway genes and one non β-oxidative pathway gene (**Figure 3**). Detailed information on the nine genes is outlined in **Supplementary Table 3**. The enzyme PAL represents the first committed step in benzenoid biosynthesis (Widhalm and Dudareva, 2015). The *LoPAL1* and *LoPAL2* genes exhibited similar expression patterns with the emission of benzoate esters (**Figure 3**), suggesting their dominant roles in the reaction of this step. The β-oxidative pathway has been elucidated in petunia flowers, and three enzymes constitute the core steps in peroxisomes, including the thioesterification of cinnamic acid by cinnamate:CoA ligase (CNL) (Colquhoun et al., 2012; Klempien et al., 2012), followed by hydration and dehydrogenation steps by cinnamoyl-CoA hydratase-dehydrogenase (CHD) (Qualley et al., 2012) and final thiolysis to produce benzoyl-CoA by 3-ketoacyl CoA thiolase (KAT) (Van Moerkercke et al., 2009). In lily, *LoCNL* expression was very low in the flower bud and highest in the blooming stage in the daytime (BM_L), while it decreased by 82.44% at night (BM_D) (**Figure 3**). The gene expression of *LoCNL* showed a close positive correlation with the emission of benzoate esters, indicating its potential role in regulating the metabolic flux towards benzenoids. The levels of *LoCHD1/2* and *LoKAT* transcripts were high in the bud stage and enhanced gradually in the BM_L and BM_D stages (**Figure 3**). To date, only benzaldehyde dehydrogenase (BALD) in the non β-oxidative pathway has been characterized in snapdragon, which accounts for the oxidation of benzaldehyde into benzoic acid (Long et al., 2009). The protein sequence of LoBALD showed 79.48% identity to snapdragon BALD (**Supplementary Table 3**). The expression of *LoBALD* could be observed in the flower bud stage and its expression increased 2.27-fold and 4.60-fold in the BM_L and BM_D stages, respectively (**Figure 3**).

Generally, methyl benzoate is synthesized from benzoic acid through the action of BAMT or benzoic acid/salicylic acid methyltransferases (BSMT), which belong to the SABATH family (D'Auria et al., 2003). Only two SABATH members were identified in the lily transcriptome. However, phylogenetic analysis showed that LoSABATH1/2 are distant from other functionally characterized plant BAMTs and BSMTs (**Supplementary Figure 1**). Furthermore, the *LoSABATH1/2* transcripts were almost undetectable in the tepals at anthesis, implying their inability to synthesize methyl benzoate in lily flowers. In case any SABATH members were omitted in our lily transcriptome, three published *L.* ‘Siberia’ flower libraries with a total of 12.4 Gb raw data (Hu et al., 2017) were added to reassemble the transcriptome using trinity software. The new assembly yielded 71,647 unigenes with an N50 length of 1,430 bp. Nevertheless, no new SABATH members were identified. In addition, we observed that Shi and colleagues identified three SABATH methyltransferases in their *L.* ‘Siberia’ transcriptome, which assembled using a total of 234.38 Gb clean data and contained 118,665 unigenes with an N50 length of 1,038 bp (Shi et al., 2018). However, all three members showed weak or no gene expression in the flowers of *L.* ‘Siberia’ (Shi et al., 2018).

### Transcriptome analysis revealed LoAAT1 as a candidate for floral ethyl benzoate formation

**Figure 4:**
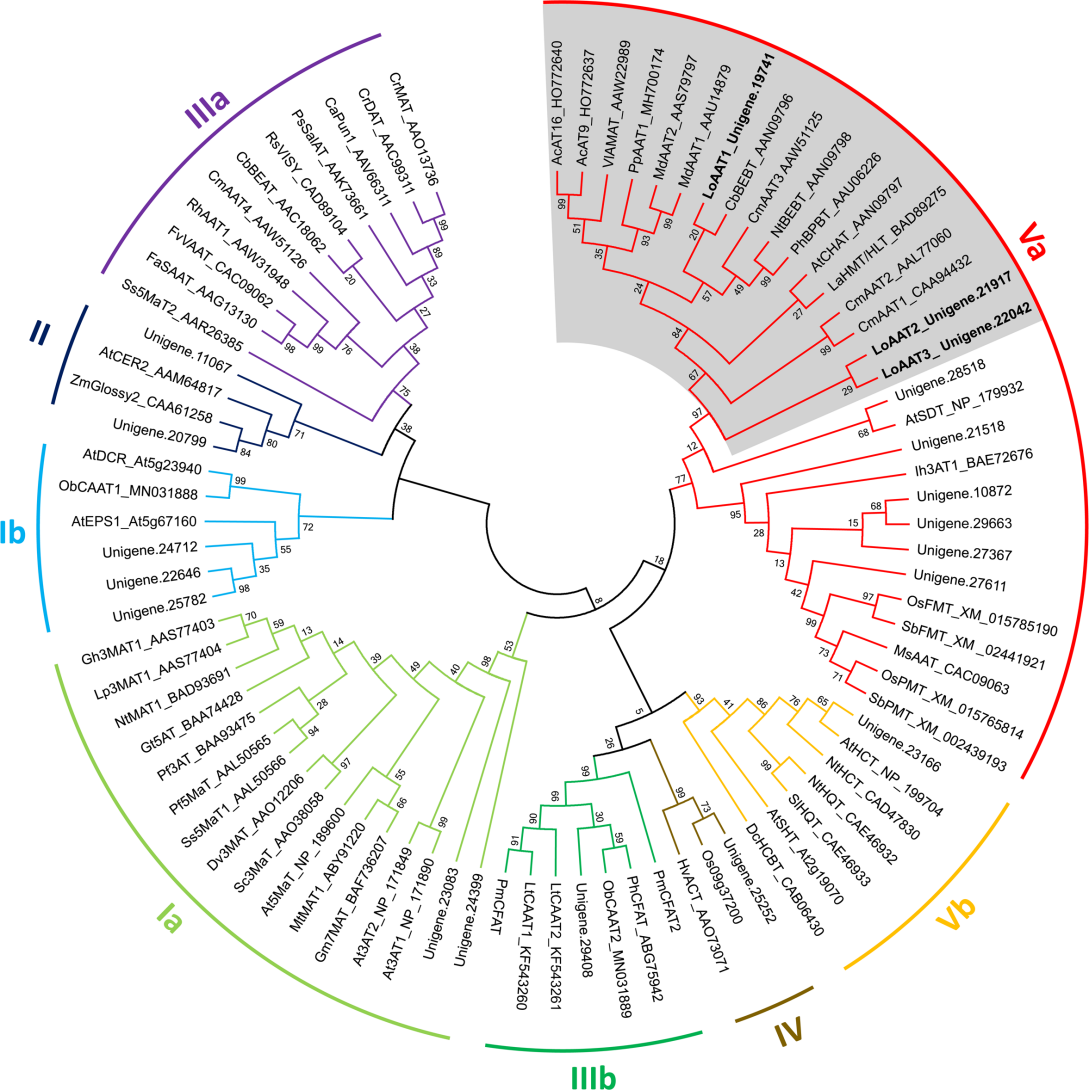
Phylogenetic analysis of BAHD members in *L.* ‘Siberia’. The phylogenetic tree was constructed based on protein sequences of functionally characterized members in the BAHD family using the neighbor-joining method. AAT orthologues in clade Va are shaded in grey. The lily BAHD members are displayed with the prefix “Unigene”. The numbers at each branch indicate bootstrap percentages from 1000 replicates. GenBank accession numbers are shown behind their corresponding enzyme name. Ac, *Actinidia chinensis*; At, *Arabidopsis thaliana*; Ca, *Capsicum annuum*; Cb, *Clarkia breweri*; Cm, *Cucumis melo*; Cr, *Catharanthus roseus*; Dc, *Dianthus caryophyllus*; Dv, *Dahlia variabilis*; Fa, *Fragaria ananassa*; Fv, *F. vesca*; Gh, *Glandularia hybrida*; Gm, *Glycine max*; Gt, *Gentiana trifloral*; Hv, *Hordeum vulgare*; Ih, *Iris hollandica*; Lo, *Lilium* oriental hybrid; Lp, *Lamium purpureum*; Lt, *Larrea tridentate*; Md, *Malus domestica*; Ms, *Musa sapientum*; Mt, *Medicago truncatula*; Nt, *Nicotiana tabacum*; Ob, *Ocimum basilicum*; Os, *Oryza sativa*; Pf, *Perilla frutescens*; Ph, *Petunia hybrid*; Pm, *Prunus mume*; Pp, *P. persica*; Ps, *Papaver somniferum*; Rh, *Rosa hybrida*; Rs, *Rauvolfia serpentine*; Sb, *Sorghum bicolor*; Sc, *Senecio cruentus*; Sl, *Solanum lycopersicum*; Ss, *Salvia splendens*; Vl, *Vitis labrusca*; Zm, *Zea mays*.

To identify the BAHD acyltransferase gene involved in the floral ethyl benzoate formation, a local BLAST search was performed using *C. breweri* CbBEBT as the query sequence (D'Auria et al., 2002). Nineteen complete BAHD acyltransferase members were identified in lily transcriptome (**Figure 4**). Phylogenetic analysis of plant BAHD acyltransferases revealed eight clades for this family (**Figure 4**, Tuominen et al., 2011). It was reported that the AATs responsible for volatile benzoate ester biosynthesis belong to clade Va (D'Auria, 2006; Tuominen et al., 2011). Among the nine members in clade Va, three fell into the group of AAT orthologues, designated LoAAT1 through LoAAT3 (**Figure 4**). However, only the *LoAAT1* gene displayed high expression in the blooming stage, and its expression exhibited a close positive correlation with the emission of ethyl benzoate (**Figure 3**), suggesting that *LoAAT1* is the top candidate gene for floral ethyl benzoate formation. Therefore, we cloned the full-length cDNA sequence of *LoAAT1*, which has a putative ORF of 1,773 bp encoding 459 amino acid residues (**Supplementary Table 3**). LoAAT1 contains the motifs HXXXD and DFGWG, which are highly conserved in almost all BAHD members (**Supplementary Figure 2**). Alignment of the amino acid sequences revealed that LoAAT1 shares 67.57% and 72.67% identity with *C. breweri* CbBEBT and petunia PhBPBT, respectively (**Supplementary Figure 2**), suggesting the possible alcohol acyltransferase function of LoAAT1.

### LoAAT1 is a methanol/ethanol benzoyl transferase

**Figure 5:**
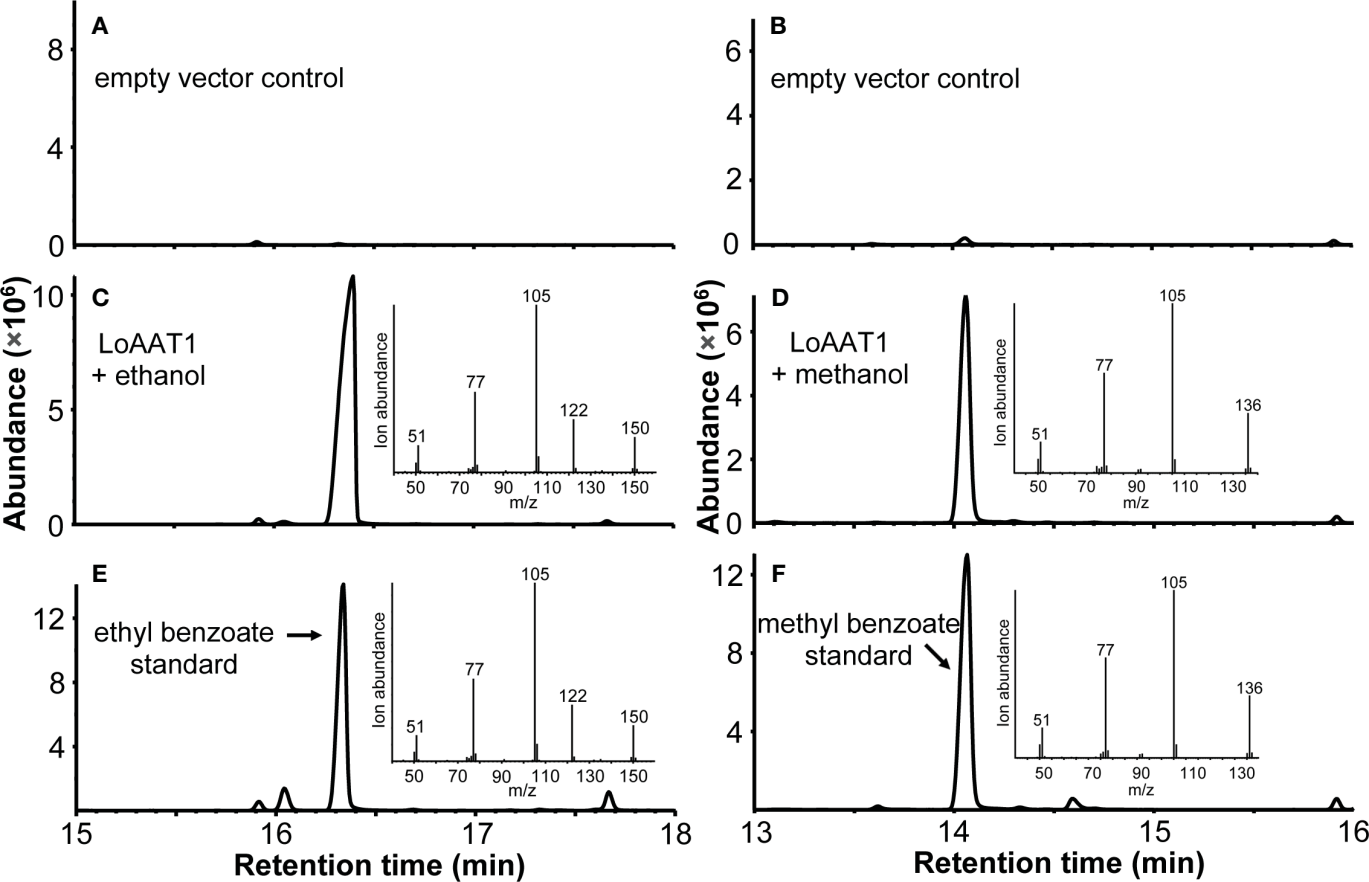
Characterization of LoAAT1 *in vitro*. **(A-D)** Total ion chromatogram (TIC) of the products generated by recombinant LoAAT1 **(C, D)** and empty vector control **(A, B)** incubated with the alcohol substrates ethanol **(A, C)** or methanol **(B, D)** using benzoyl-CoA as the acyl donor. **(E, F)** TIC of ethyl benzoate **(E)** and methyl benzoate **(F)** authentic standards. Insets in panels **(C-F)** represent the mass spectra of the corresponding main peaks.

To characterize the function of LoAAT1, the coding region was expressed in *Escherichia coli*, and its acyltransferase activity was tested using benzoyl-CoA/hexyl-CoA and various alcohol substrates *in vitro*. The results showed that LoAAT1 could convert ethanol and benzoyl-CoA into ethyl benzoate (**Figure 5**). Interestingly, it could also catalyze methanol and benzoyl-CoA to produce methyl benzoate (**Figure 5**). With benzoyl-CoA as the acyl donor, a high level of benzoylated products was observed when butanol and 2-hexanol were offered as alcohol substrates for LoAAT1, and fewer benzoylated products were detected with the alcohol substrates octanol, 2-phenethyl alcohol and benzyl alcohol (**Supplementary Figure 3**). When monoterpene alcohol served as a substrate, only a slight level of catalytic activity was observed towards geraniol, while no activity towards linalool was observed (**Supplementary Figure 4**). In addition, LoAAT1 could also utilize hexyl-CoA as the acyl donor with high catalytic activity towards the above alcohol substrates, except for lower activity with methanol and 2-phenethyl alcohol (**Supplementary Figure 5**). Likewise, LoAAT1 could not acylate linalool with hexyl-CoA as the acyl donor (**Supplementary Figure 6**).

To confirm the acyltransferase activity in lily flowers, various alcohol substrates were fed through the infiltration method (Yue et al., 2021). After the infiltration of alcohol substrates, including butanol, 2-hexanol, octanol, 2-phenethyl alcohol, benzyl alcohol, geraniol and linalool, their corresponding benzoylated products were newly added into the floral scent profiles of *L.* ‘Siberia’, except linalool, exhibited identical substrate specificity to the LoAAT1 protein with benzoyl-CoA *in vitro* (**Supplementary Figure 7**). The results indicated that the benzoyltransferase activity in lily flowers might function dominantly by LoAAT1. Increasing the supply of ethanol in tepals significantly increased the level of ethyl benzoate and decreased methyl benzoate emission. Infiltration of equal amounts of ethanol and methanol also increased the ethyl benzoate release and reduced the emission of methyl benzoate (**Supplementary Figure 8**), implicating an abundant methanol pool and small ethanol pool in tepals. However, sufficient butanol supply resulted in a significant decline in both methyl benzoate and ethyl benzoate releases and a massive emission of newly generated butyl benzoate (**Supplementary Figure 8**). The results showed that LoAAT1 is a methanol/ethanol benzoyl transferase *in planta*, and alcohol substrate availability and its pool size are limiting factors for the formation of floral benzoate esters.

### Tepal-specific, floral development-regulated and rhythmic expression of the *LoAAT1* gene

**Figure 6:**
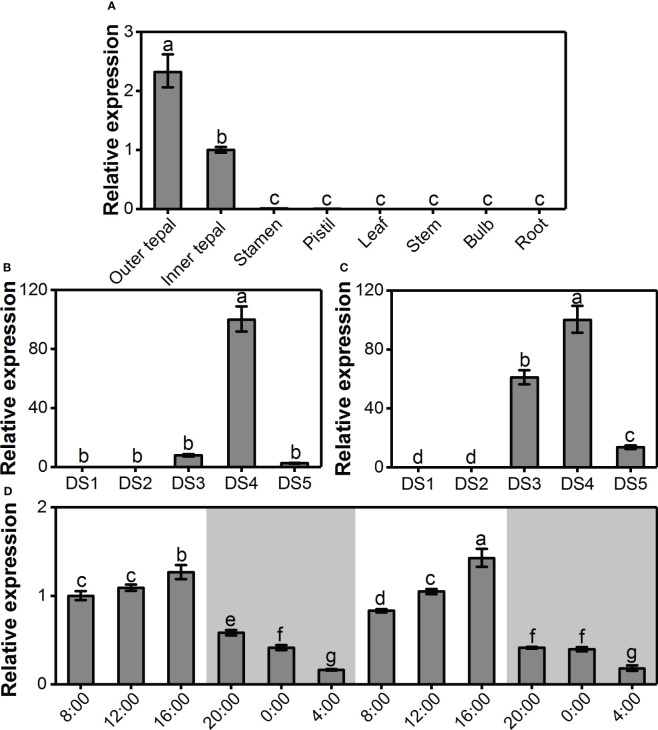
Tepal-specific, floral development-regulated and rhythmic expression of the *LoAAT1* gene in *L.* ‘Siberia’. **(A)** The expression pattern of *LoAAT1* in different tissues. **(B, C)** Expression analysis of *LoAAT1* in the outer **(B)** and inner tepals **(C)** at different floral developmental stages. **(D)** Changes in *LoAAT1* transcripts in tepals during two consecutive light/dark (12-h/12-h) cycles on the day after full-opening. White and grey areas correspond to light and dark, respectively. Error bars indicate the calculated maximum and minimum expression quantity of three replicates. Different lowercase letters labelled on bars indicate statistically significant differences at the level of *P* < 0.05.

To reveal the correlation of *LoAAT1* gene expression with benzoate ester formation, the spatiotemporal and rhythmic expression levels of *LoAAT1* were assessed by real-time PCR. Coinciding with the emission of methyl benzoate and ethyl benzoate, *LoAAT1* was only expressed in the outer tepals and inner tepals, exhibiting a tepal-specific expression pattern (**Figure 6A**). During flower development, *LoAAT1* transcripts were almost undetectable in outer and inner tepals at the bud stages of DS1 and DS2 (**Figure 6B, C**). In the process of flower opening, the expression of *LoAAT1* increased promptly in inner tepals, reached a maximum level at the full-opening stage (DS4) and then declined to a low level in the senescence stage (DS5, **Figure 6C**). For outer tepals, the highest expression level of *LoAAT1* was also observed at the full-opening stage, with weak expression levels in the DS3 and DS5 stages (**Figure 6B**). Notably, *LoAAT1* expression was developmentally regulated, showing a positive correlation with benzoate ester release. The gene expression of *LoAAT1* displayed identical rhythmic changes during two daily light/dark cycles in the tepals at anthesis (**Figure 6D**). *LoAAT1* transcripts increased gradually during the day (8:00 to 16:00), reached a maximum at 16:00, decreased at night (2:00 to 4:00), and reached a minimum at 4:00 (**Figure 6D**), displaying similar fluctuation with benzoate ester emissions.

### Analysis of floral benzoate ester emission and *AAT1* expression in different lily varieties

**Figure 7:**
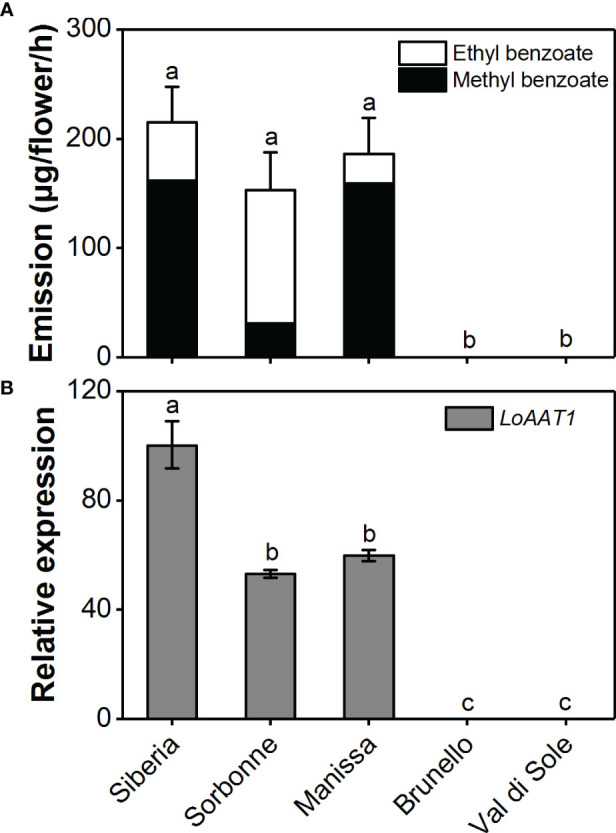
Floral benzoate ester emission **(A)** and *LoAAT1* orthologous expression **(B)** in scented and nonscented *Lilium* species. Relative expression level of *LoAAT1* in tepals of *L.* ‘Siberia’ was set to 1 (100%). Different lowercase letters labelled on bars indicate statistically significant differences at the level of *P* < 0.05 (n=3).

The emission variation of floral benzoate esters in the scented *Lilium* Oriental hybrids ‘Siberia’ and ‘Sorbonne’, the Oriental × Trumpet (OT) hybrid ‘Manissa’, and the nonscented Asiatic hybrids ‘Brunello’ and ‘Val di Sole’ was investigated using headspace collection and GC-MS analysis (**Figure 7A**). In addition to ‘Siberia’, the scented flowers of ‘Sorbonne’ and ‘Manissa’ also released high amounts of benzoate esters. Contrary to ‘Siberia’ and ‘Manissa’, ‘Sorbonne’ released more ethyl benzoate relative to methyl benzoate. As expected, there were no benzoate esters emitted from the non-scented flowers of ‘Brunello’ and ‘Val di Sole’. To unveil the mechanism underlying the variation in floral benzoate esters emission among scented and nonscented *Lilium* species, the expression levels of *LoAAT1* orthologues were measured using real-time PCR (**Figure 7B**). The results showed that *AAT1* was highly expressed in scented species and no *AAT1* transcript was detected in nonscented species, suggesting the pivotal role of adequate *AAT1* expression for floral benzoate ester emission in lily. Furthermore, feeding different alcohol substrates to scented ‘Sorbonne’ and ‘Manissa’ tepals also transformed the floral benzoate ester profiles (data not shown). Thus, the gene expression of *AAT1* orthologues and substrate availability contribute to the floral benzoate ester production in lily.

## Discussion

### LoAAT1 is responsible for the biosynthesis of floral ethyl benzoate

Volatile esters are a major class of compounds contributing to floral scent and fruit aroma, such as geranyl/citronellyl acetate in rose and hexenyl acetate in apricot (Shalit et al., 2003; Zhou et al., 2021). The biosynthesis of these compounds is usually catalyzed by AAT in the BAHD family (St-Pierre and De Luca, 2000; D'Auria, 2006). To date, only a small number of AATs accounting for the formation of floral volatile esters have been characterized, especially for floral benzoate esters. *C. breweri* BEAT is the first isolated and functionally characterized plant AAT, which catalyzes the formation of floral benzylacetate, a major constituent of the *C. breweri* floral scent (Dudareva et al., 1998). In petunia, the alcohol acyltransferase BPBT was confirmed to produce volatile benzoate esters benzylbenzoate and phenylethyl benzoate in corollas (Boatright et al., 2004). Ethyl benzoate is one of the dominant floral volatiles in some scented lily cultivars (Kong et al., 2012). However, its biosynthesis remains unclear. In this study, we identified a BAHD acyltransferase gene, *LoAAT1*, which exhibits a high gene expression in the tepals, and its protein sequence has the highest identity with the functionally characterized benzoyltransferases among the three AAT homologues identified in *L.* ‘Siberia’ (**Figures 3**, **4**). *In vitro* enzyme assays showed that LoAAT1 can synthesize ethyl benzoate using the substrates ethanol and benzoyl-CoA (**Figure 5**). In addition, LoAAT1 could accept a diverse range of alcohol substrates with discrepant catalytic activity *in vitro*, which showed identical alcohol substrate preference to the acyltransferases in flowers (**Figure 5**, **Supplementary Figures 3, 4, 7**), suggesting that the benzoyltransferase activity *in planta* might function dominantly by LoAAT1. Furthermore, increasing the supply of ethanol in tepals through the infiltration method significantly increased the level of ethyl benzoate, while increasing butanol supply resulted in a significant decline for ethyl benzoate release, implicating that ethanol is a substrate for LoAAT1 *in planta* (**Supplementary Figure 8**). Taken above evidences together, we concluded that LoAAT1 is responsible for the biosynthesis of floral ethyl benzoate in *L.* ‘Siberia’. In kiwifruit, the volatile ester ethyl benzoate was also formed through the benzoylation of ethanol by an AAT in BAHD family (Gunther et al., 2011).

### Contribution of LoAAT1 to the biosynthesis of floral methyl benzoate

Methyl benzoate is a common ingredient of floral volatiles, and often emerges in the floral scent profiles of fragrant flowers, such as lily (Du et al., 2019), tuberose (Kutty and Mitra, 2019), *N. suaveolens* (Raguso et al., 2003), and *H. coronarium* (Yue et al., 2021). In addition to its fruity odor contributing to floral fragrance for humans, methyl benzoate often acts as a volatile signal to recruit pollinators for reproduction (Kessler et al., 2013). The biosynthesis of methyl benzoate has been well investigated in several plants, including snapdragon (Dudareva et al., 2000), petunia (Negre et al., 2003), *N. suaveolens* (Pott et al., 2004), and *H. coronarium* (Yue et al., 2021). To date, methyl benzoate in most plants has been reported to be synthesized from benzoic acid and *S*-adenosyl-L-methionine by the action of BAMT or BSMT of the SABATH family (Effmert et al., 2005; Yue et al., 2021). In *Lilium* ‘Yelloween’, the BSMT gene *LiBSMT* was reported to be involved in floral methyl benzoate production (Wang et al., 2015). Although methyl benzoate is one of the main compounds in floral scent profile of *L.* ‘Siberia’, we did not identify SABATH methyltransferase candidate gene that possessed of adequate gene expression level for methyl benzoate biosynthesis in the transcriptome of *L.* ‘Siberia’ (**Figure 3**). This finding is consistent with another research on the transcriptome of flowers in *L.* ‘Siberia’, and in which the orthologue of *LiBSMT* in *L.* ‘Siberia’ exhibited no expression in the flowers (Shi et al., 2018; S. Shi, personal communication).

It is reported that overexpressing apple AAT gene *MdAAT2* in transgenic tobacco leaves led to the production of methyl caprylate, methyl caprate, and methyl dodecanoate, and significantly increased the concentrations of methyl benzoate and methyl tetradecanoate (Li et al., 2008), suggesting the possible roles of plant AAT in the formation of methyl esters, including methyl benzoate. Interestingly, we found that LoAAT1 can catalyze the formation of methyl benzoate using methanol and benzoyl-CoA as substrates (**Figure 5**). Moreover, the gene expression pattern of *LoAAT1* is similar with the emission of methyl benzoate (**Figure 6**). In addition, the infiltrations of exogenous ethanol or butanol into tepals led to the significant decrease of floral methyl benzoate (**Supplementary Figure 8**), implying the competition of exogenous alcohols with the endogenous menthol for the benzoyltransferase activity of LoAAT1. Collectively, we conclude that LoAAT1 contributes to the biosynthesis of the floral scent compound methyl benzoate in *L.* ‘Siberia’, representing an alternative mechanism for methyl benzoate formation in plant. To our knowledge, a few floral volatiles can be synthesized by enzymes in distinct protein families. For example, methyl anthranilate, a compound involved in fruit aroma and plant defense, has also been shown to be formed either by acyltransferase or methyltransferase (Wang and De Luca, 2005; Kollner et al., 2010; Pillet et al., 2017). Grape alcohol acyltransferase AMAT catalyzes the formation of methyl anthranilate from methanol and anthraniloyl-CoA (Wang and De Luca, 2005), while maize methyltransferase AAMT1 contributes to the conversion of anthranilic acid into methyl anthranilate (Kollner et al., 2010), indicating dual biosynthetic mechanisms for one volatile compound.

Methanol, as the simplest alcohol in nature, is generally produced in the reaction of demethylation of macromolecules, including DNA and proteins. In plants, the main source of methanol derives from the demethylesterification reaction of pectins in the cell wall, which are directed by pectin methylesterases (PMEs) (Dorokhov et al., 2018). PMEs play a crucial role in plant growth and development processes including cell wall extension and stiffening, pollen formation, and fruit ripening (Pelloux et al., 2007). Interestingly, we identified a highly expressed *LoPME* gene in the flowers of lily, whose gene expression displayed a close positive correlation with the emission of methyl benzoate (**Supplementary Table 3**), indicating a synergetic methanol supply for LoAAT1 to produce floral methyl benzoate.

### Regulation of floral benzoate ester biosynthesis in lily

Although LoAAT1 has wide alcohol substrate specificity, only massive methyl benzoate and little ethyl benzoate were present in the scent profile of *L.* ‘Siberia’. This observation might be due to the abundant methanol and small ethanol internal pool in tepals, as well as the absence of an internal pool for other alcohols. This speculation could be verified by the infiltration assays of exogenous alcohols into tepals, which caused the release of corresponding benzoate esters and decreased the emission of methyl benzoate (**Supplementary Figures 7, 8**). In contrast, despite the existence of linalool in flowers, its benzoylated product could not be detected in floral volatiles, because of the inability of LoAAT1 to catalyze linalool (**Supplementary Figure 4**). Thus, the substrate availability, pool size and specificity of LoAAT1 might determine the floral benzoate ester profiles in *L.* ‘Siberia’. This scenario is also applicable in other flowers. The petunia BPBT protein can use a broad range of alcohols in the presence of benzoyl-CoA and has a comparable catalytic efficiency with benzyl alcohol and phenylethanol. However, the product pool of benzyl benzoate is 14-fold larger than that of phenylethyl benzoate, resulting from the twenty times larger amount of benzyl alcohol relative to phenylethanol available to the enzyme (Boatright et al., 2004). By the way, the wide substrate specificity of LoAAT1 towards alcohols reminds us to avoid any exogenous alcohols in the headspace environment when measuring floral volatile compounds in lily in the case of alteration of the floral scent profile.

The emission of floral volatiles often occurs in a flower-specific, developmentally regulated and sometimes rhythmic manner (Dudareva et al., 2013). Methyl benzoate in snapdragon is released from only the upper and lower lobes of petals, and is developmentally regulated in flowers. Its emission exhibits a circadian rhythmic pattern, with maximum emission during the day, which coincides with the activity of pollinator bumblebees (Kolosova et al., 2001). In the flowers of *L.* ‘Siberia’, the emission of methyl benzoate and ethyl benzoate displayed a tepal-specific pattern and was regulated by floral development, with maximum emission at the full-opening stage (**Figure 1**). In addition, their emission occurred in a rhythmic manner and peaked at 16:00, which is the most fragrant period in lily flowers (**Figure 2**). Meanwhile, the gene expression of *LoAAT1* also exhibited tepal-specific, floral development-regulated and rhythmic patterns and showed a positive correlation with the emission of benzoate esters, indicating the critical role of *LoAAT1* in regulating the biosynthesis of floral benzoate esters in *L.* ‘Siberia’. In petunia, the alcohol acyltransferase BPBP is responsible for the biosynthesis of both benzyl benzoate and phenylethyl benzoate in flowers. Its transcript levels are closely correlated with the pattern of BPBT activity and benzyl benzoate accumulation (Boatright et al., 2004), suggesting the dominant roles of *AAT* gene expression in the spatiotemporal and rhythmic regulation of floral benzoate ester emission.

In addition to the AAT gene expression and alcohol substrates, the supply of the acyl donor also affects the biosynthesis of floral volatile esters (D'Auria et al., 2002). Although hexyl-CoA is available for LoAAT1 *in vitro*, no hexylated product was detectable in the floral scent profiles of lily (**Supplementary Figures 5-7**), implying a shortage of the hexyl-CoA pool in lily flowers. Benzoyl-CoA/benzoic acid, the precursor for volatile benzenoids, is produced via both β-oxidative and non β-oxidative pathways in petunia flowers (Boatright et al., 2004). Benzaldehyde is a key metabolic intermediate in the non-β-oxidative pathway and is one of the major floral volatile compounds in petunia (Boatright et al., 2004). However, no benzaldehyde was detected in the floral scent profile of *L.* ‘Siberia’, suggesting the dominant role of the β-oxidative pathway in supplying benzoyl-CoA for floral benzenoid biosynthesis in lily. In the β-oxidative pathway of lily, the gene expression of *LoCNL* showed a close positive correlation with the emission of benzoate esters, indicating that *LoCNL* seems to be the key gene in regulating the supply of benzoyl-CoA donors (**Figure 3**). Recently, CNL was considered an evolutionary hotpot for the trait of floral benzenoids. In the genus *Capsella*, inactivated CNL1 caused by point mutations contributes to the loss of floral scent during the transition from outbreeding to selfing (Sas et al., 2016). Loss of CNL function for premature termination of protein translation in petunia results in the absence of floral scent in the evolution of hummingbird-adapted species (Amrad et al., 2016). Accordingly, in addition to *LoAAT1*, *LoCNL* is also a promising target when engineering floral benzenoid traits in lily. In addition, other genes in the benzenoid biosynthetic pathway, *LoPAL1/2*, *LoCHD1*, and *LoKAT*, also possessed higher expression at anthesis relative to the floral bud (**Figure 3**), exhibiting coordinated expression in the formation of floral benzenoids. The synchronized regulation of benzenoid biosynthetic pathway genes was also observed in the flowers of petunia and *H. coronarium* (Yue et al., 2021).

Although floral benzenoid profiles vary in different lily cultivars, methyl benzoate and/or ethyl benzoate are often the major benzenoids in scented lily (Kong et al., 2012; Kong et al., 2017). They were present in the scented lily cultivars and were absent in nonscented cultivars (**Figure 7A**). As expected, the expression of *AAT1* orthologues was high in scented cultivars and weak in nonscented cultivars (**Figure 7B**), suggesting the pivotal role of *AAT1* gene expression in the formation of floral benzoate esters in lily. Moreover, the feeding assays of alcohol substrates into the tepals of other scented cultivars revealed alcohol substrate availability as another limiting factor for the biosynthesis of floral benzoate esters in lily. In view of the LiBSMT that was reported to be involved in floral methyl benzoate production in *Lilium* ‘Yelloween’ (Wang et al., 2015), the contribution of two distinct biosynthetic mechanisms for methyl benzoate remains to be demonstrated in other lily cultivars. In addition, the effect of biosynthetic pathway genes on floral benzenoid emission in other scented lily cultivars should be investigated in the future.

## Conclusion

Through the combination of volatile profiling, transcriptome profiling, *in vitro* and *in planta* assays, we identified and characterized a novel BAHD acyltransferase, namely LoAAT1, that contributes to the biosynthesis of both ethyl benzoate and methyl benzoate in the flowers of scented Lilium oriental hybrid ‘Siberia’. Biosynthesis of methyl benzoate in lily that utilizes a BAHD acyltransferase using methanol and benzoyl-CoA as substrates is in contrast to the biosynthesis of this widely occurring volatile compound in other plants, where a member of the SABATH methyltransferase family is responsible. Our results provide new insights into the molecular mechanism of benzoate esters biosynthesis in plant and pinpoint useful targets for genetic modification of scent-related traits in lily.

## Materials and methods

### Plant materials

*Lilium* ‘Siberia’ was grown in a horticulture chamber in South China Agricultural University (Guangzhou, China) under natural light from March to June. The lily cut flowers used for volatile analysis and gene expression analysis in different flower developmental stages, different sampling times, and different cultivars were purchased from the Lingnan flower market in Guangzhou. Flowers with three buds were selected and immediately placed in tap water after being brought back to the laboratory. The flower buds with ~2 cm pedicels were separated two days ahead of anthesis and cultured in a light incubator at 25°C under 12-h light and 12-h dark cycles.

### Headspace collection and GC-MS analysis

The *L.* ‘Siberia’ plants at anthesis were divided into eight tissues. Each tissue was detached carefully at its junctions, and approximately 3 g of tissue sample was enclosed in a 200-ml glass bottle for headspace collection. For analysis of benzoate ester emissions in different flower developmental stages, different sampling times, and different cultivars, one intact flower or flower bud with an ~2 cm pedicel was enclosed in an 8-L glass chamber for headspace collection. For alcohol substrate feeding, various alcohol substrates with excess amounts for catalytic reaction (2 mM each) were infiltrated into an inner tepal by a needleless syringe as previously performed (Yue et al., 2021). The infiltrated tepal was immediately enclosed in a 200-ml glass bottle for headspace collection. Headspace collection and GC-MS analysis were conducted as previously described (Abbas et al., 2019; Yue et al., 2021). Briefly, a SPME fiber (Supelco) was used to adsorb volatiles for 30 min after equilibrium of volatiles and internal standard (1.728 μg ethyl caprate) for 30 min. Then, trapped volatiles were analyzed by a GC-MS system with Agilent 7890A GC and Agilent 5975C MSD. Separation was performed on an Agilent HP-5MS capillary column (30 m × 0.25 mm) with helium as the carrier gas at a constant flow rate of 1 ml/min. The temperature program was as follows: 40°C for 2 min, followed by a ramp of 5°C/min to 100°C, 10°C/min to 190°C, 60°C/min to 250°C, and finally held at 250°C for 5 min. The volatiles were identified by comparing the retention times and mass spectra with authentic standards. The relative quantification of volatile benzoate esters was calculated using Agilent ChemStation Data Analysis Application based on the peak area ratio and the quantity of internal standard. Analysis of variance was conducted by SPSS software using Tukey’s test (*P* = 0.05).

### Transcriptome sequencing

Total RNA of *L.* ‘Siberia’ tepals in the bud stage and the blooming stage at 16:00 (BM_L) and 4:00 (BM_D) were extracted using a HiPure Plant RNA Mini Kit (Magen) according to the manufacturer’s instructions. The transcriptome sequencing library was pooled by mixing equal quantities of total RNA from the three samples. For transcript quantification, three libraries were generated independently for the above three samples. The sequencing libraries were prepared using an Illumina TruSeq RNA Sample Preparation Kit and sequenced on an Illumina HiSeq 2000 platform as described previously (Yue et al., 2015). The 100 bp paired-end reads were generated for transcriptome assembly, and 100 bp single-end reads were generated for transcript quantification. After raw reads were filtered, the high-quality clean reads were used to assemble the lily transcriptome with Trinity software using the default parameters for *de novo* assembly without a reference genome (Grabherr et al., 2011). All assembled unigene sequences were annotated against public databases as described previously (Yue et al., 2015). For transcript quantification, the clean reads of bud, BM_L and BM_D libraries were mapped to the assembled lily reference transcriptome, and then the readcount of each unigene was estimated using RSEM software (Li and Dewey, 2011). Subsequently, readcount was normalized to RPKM (reads per kilobase of exon model per million mapped reads) (Mortazavi et al., 2008). Differential expression analysis between two samples was performed using the DEGseq R package (Wang et al., 2010). The absolute value of log_2_(fold change) > 1 and *q* value < 0.005 was set as the threshold to judge the differentially expressed genes (DEGs).

### Lily SABATH and BAHD identification and phylogenetic analysis

To identify the SABATH and BAHD members in the lily transcriptome, the functionally characterized rice OsBSMT (Zhao et al., 2010) and *C. breweri* CbBEBT (D'Auria et al., 2002) were used as query sequences for a local blast search with an E-value threshold of 10^-5^. The candidate genes were confirmed using their conserved motifs by NCBI CD-search and Pfam software (Marchler-Bauer et al., 2017; Mistry et al., 2021). The phylogenetic tree was constructed with MEGA7 using the neighbor-joining method after the amino acid sequences were aligned with ClustalW.

### Amplification of full-length *LoAAT1* gene

The full-length cDNA sequence of *LoAAT1* was amplified using high-fidelity DNA polymerase KOD-Plus (TOYOBO) with the primers listed in **Supplementary Table 4**. The alignment was performed by ClustalX and shaded with GeneDoc.

### Bacterial expression and purification of LoAAT1 recombinant protein

The coding sequence of *LoAAT1* was amplified using high-fidelity DNA polymerase KOD-Plus (TOYOBO) with the primers in **Supplementary Table 4** and then constructed into the pET30a vector (Novagen) through the cloning sites *Eco*RI and *Hind*III. The sequencing results showed that no errors had been introduced. The recombinant vectors were transformed into *E. coli* Rosetta (DE3) competent cells (Invitrogen) for recombinant protein induction as described previously (Yue et al., 2014). His-tagged recombinant protein was purified with Ni-NTA His·Bind Resins (Novagen) following manufacturer’s introductions. The pET30a empty vector was used as a negative control.

### *In vitro* enzyme assay of LoAAT1

Enzyme assays were conducted in 5-ml sealed glass vials with a total volume of 1 ml consisting of 50 mM Tris-HCl, pH 7.0, 20 μg partially purified LoAAT1 protein, 0.1 mM benzoyl-CoA or hexyl-CoA, and 0.5 mM alcohol substrates (methanol, ethanol, butanol, 2-hexanol, octanol, 2-phenethylalcohol, benzyl alcohol, geraniol, or linalool). The mixture was incubated at 28°C for 30 min, and then the volatile products were trapped by a SPME fiber for 30 min and identified through a GC-MS system. The GC temperature was initially maintained at 40°C for 2 min, followed by an increase to 250°C at a rate of 5°C/min, and held at 250°C for 5 min. The reaction products were validated by comparing the retention times and/or mass spectra with authentic standards.

### Real-time PCR

The samples were prepared as described for headspace collection. Total RNA was extracted as described above, and then reverse transcribed using the PrimeScript RT reagent Kit with gDNA Eraser (TaKaRa) according to the manufacturer’s instructions. The sequence-specific primers for *LoAAT1* and the reference gene *LoGAPDH* are listed in **Supplementary Table 4**. Real-time PCR was performed on an ABI 7500 Real-Time PCR System using iTaq Universal SYBR Green Supermix (Bio-Rad) as described previously (Yue et al., 2015; Abbas et al., 2019). Three independent amplifications were performed for each sample. The relative gene expression of *LoAAT1* was calculated according to the 2^-ΔΔCt^ method (Livak and Schmittgen, 2001). Analysis of variance was performed by SPSS software using Tukey’s test (P = 0.05).

## Data availability statement

The datasets presented in this study can be found in online repositories. The names of the repository/repositories and accession number(s) can be found below: https://www.ncbi.nlm.nih.gov/genbank/, OR288168 https://www.ncbi.nlm.nih.gov/genbank/, PRJNA996359.

## Author contributions

YCY: Conceptualization, Data curation, Formal Analysis, Investigation, Visualization, Writing – original draft. LW: Formal Analysis, Investigation, Writing – original draft. ML: Formal Analysis, Investigation, Writing – original draft. FL: Investigation, Writing – original draft. JY: Investigation, Writing – original draft. LH: Investigation, Writing – original draft. BZ: Data curation, Writing – original draft. XL: Formal Analysis, Writing – original draft. YYY: Formal Analysis, Writing – original draft. FC: Writing – review & editing. RY: Writing – review & editing, Funding acquisition, Supervision. YF: Conceptualization, Funding acquisition, Project administration, Writing – original draft.
