# Supplementary material for: A BAHD acyltransferase contributes to the biosynthesis of both ethyl benzoate and methyl benzoate in the flowers of Lilium oriental hybrid ‘Siberia’
Source: Front Plant Sci. 2023 Sep 29;14:1275960. doi: 10.3389/fpls.2023.1275960 (PMC10570747; doi:10.3389/fpls.2023.1275960)
Supplement: Supplementary file 1 [file DataSheet_1.pdf]

Supplementary Materials

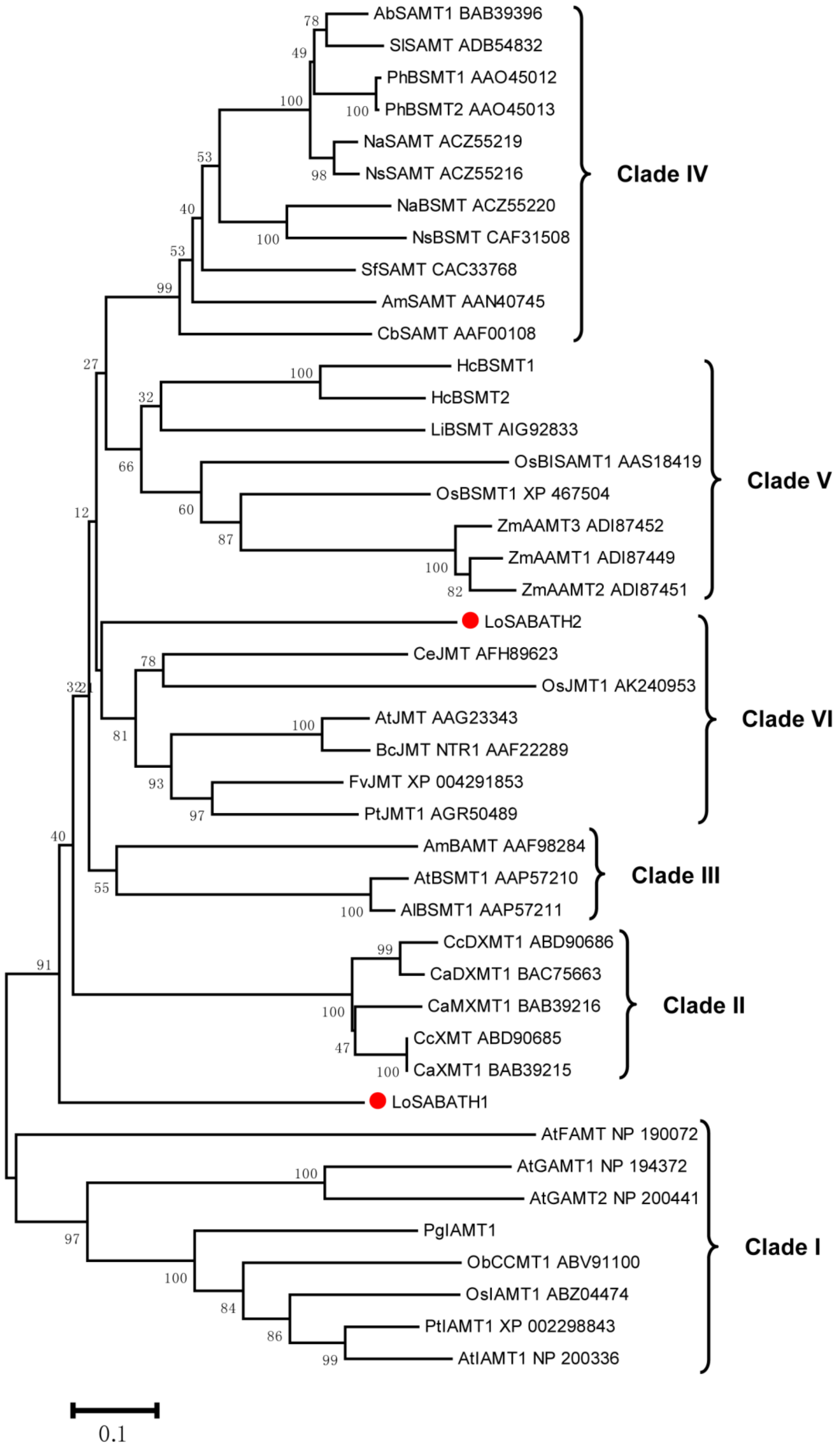

Supplementary Figure 1. Phylogenetic analysis of SABATHs in *Lilium* 'Siberia'. The phylogenetic tree was constructed based on protein sequences of functionally characterized members in the SABATH family using the neighbour-joining method. The members of the plant SABATH family are classified into six clades (Clade I through Clade VI). The SABATH proteins identified from the lily transcriptome are labelled with red solid circles. The scale bar indicates 10% sequence divergence. The numbers at each branch indicate bootstrap percentages from 1,000 replicates. GenBank accession numbers are shown behind their corresponding enzyme name. Ab, *Atropa belladonna*; Al, *Arabidopsis lyrata*; Am, *Antirrhinum majus*; At, *A. thaliana*; Bc, *Brassica campestris*; Ca, *Coffea arabica*; Cb, *Clarkia breweri*; Cc, *C. canephora*; Ce, *Cymbidium ensifolium*; Fv, *Fragaria vesca*; Hc: *Hedychium coronarium*; Li, *Lilium* 'Yelloween'; Lo: *Lilium* oriental hybrid; Na, *Nicotiana alata*; Ns, *N. suaveolens*; Ob, *Ocimum basilicum*; Os, *Oryza sativa*; Pg, *Picea glauca*; Ph, *Petunia hybrid*; Pt, *Populus trichocarpa*; Sf, *Stephanotis floribunda*; Sl, *Solanum lycopersicum*; Zm, *Zea mays*.

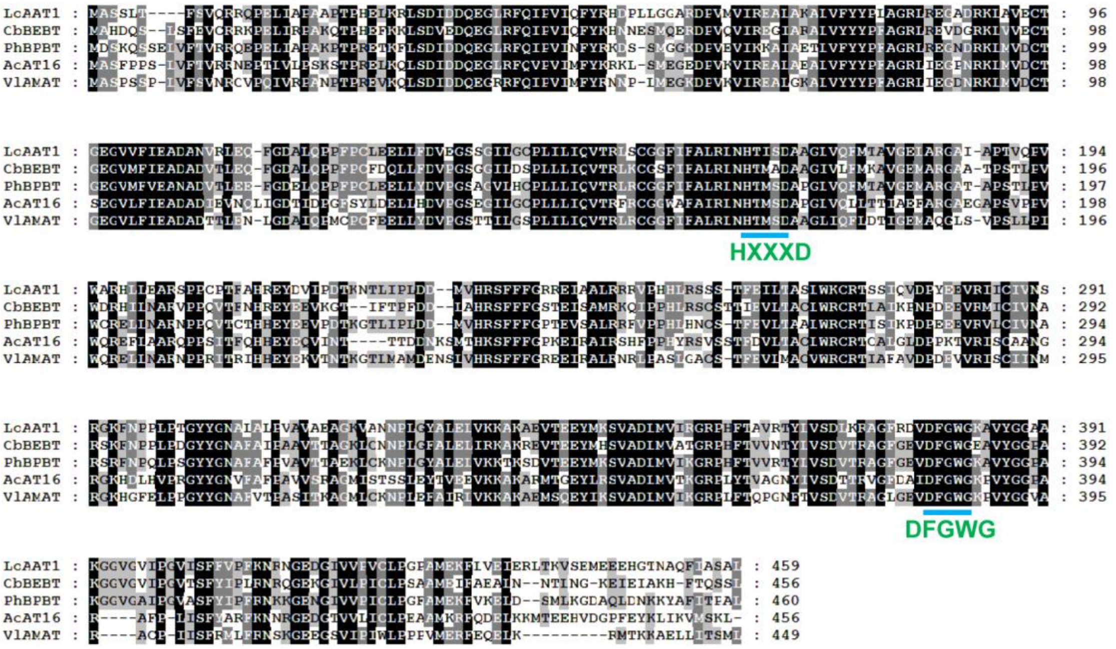

Supplementary Figure 2. Alignment of the amino acid sequence of LoAAT1 with representative alcohol acyltransferases. Amino acid residues shaded in black, grey and light grey represent 100, 80 and 60% conserved identity, respectively. Dashes indicate gaps inserted for optimal alignment. The conserved HXXXD and DFGWG motifs of the BAHD family are underlined.

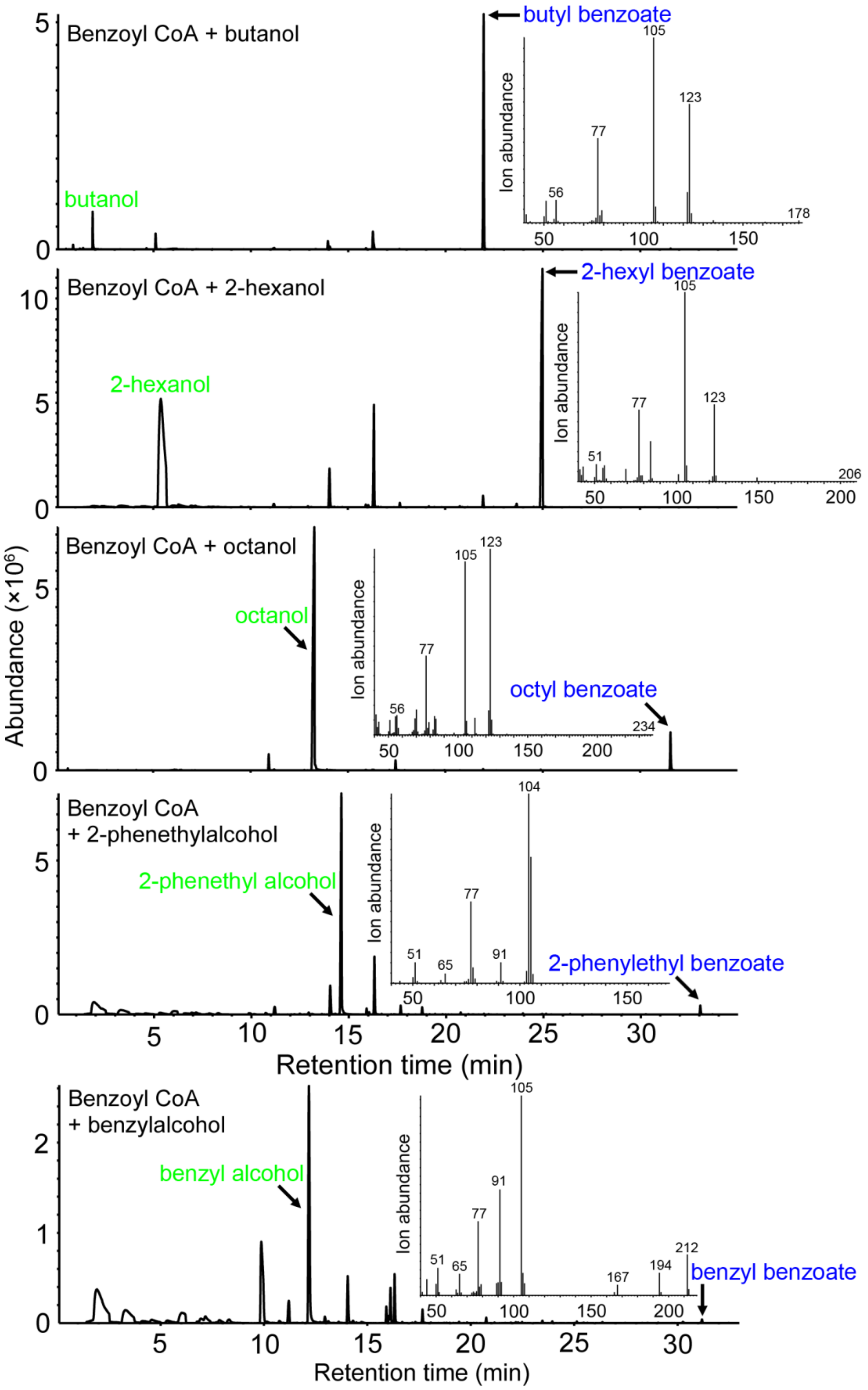

Supplementary Figure 3. GC-MS analysis of products generated by recombinant LoAAT1 incubated with benzoyl-CoA and different alcohol substrates. The alcohol substrates are shown in green, while the benzoylated products whose mass spectra are displayed as insets are shown in blue.

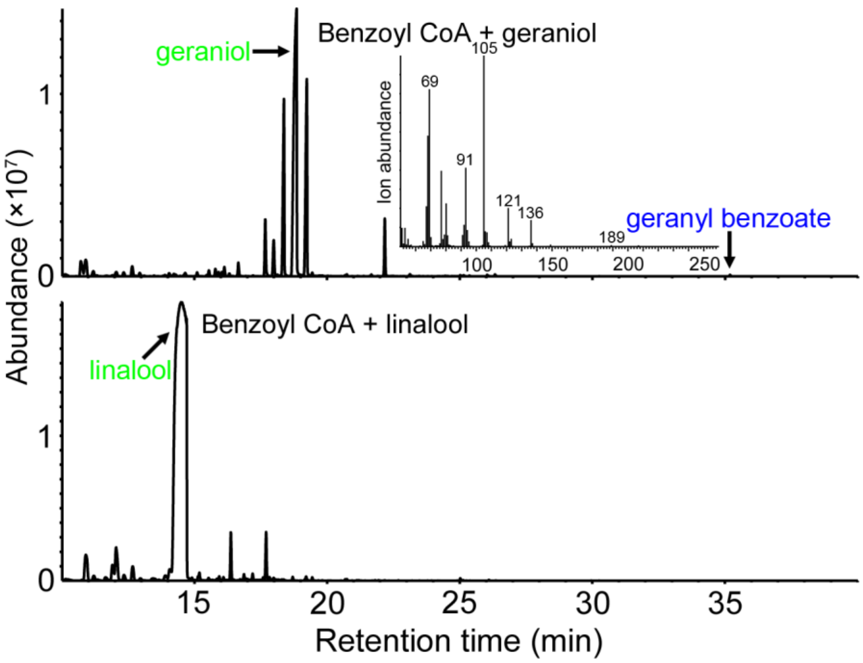

Supplementary Figure 4. GC-MS analysis of products generated by recombinant LoAAT1 incubated with benzoyl-CoA and the monoterpene alcohol substrates. The alcohol substrates are shown in green while the benzoylated product whose mass spectrum is displayed as an inset is shown in blue.

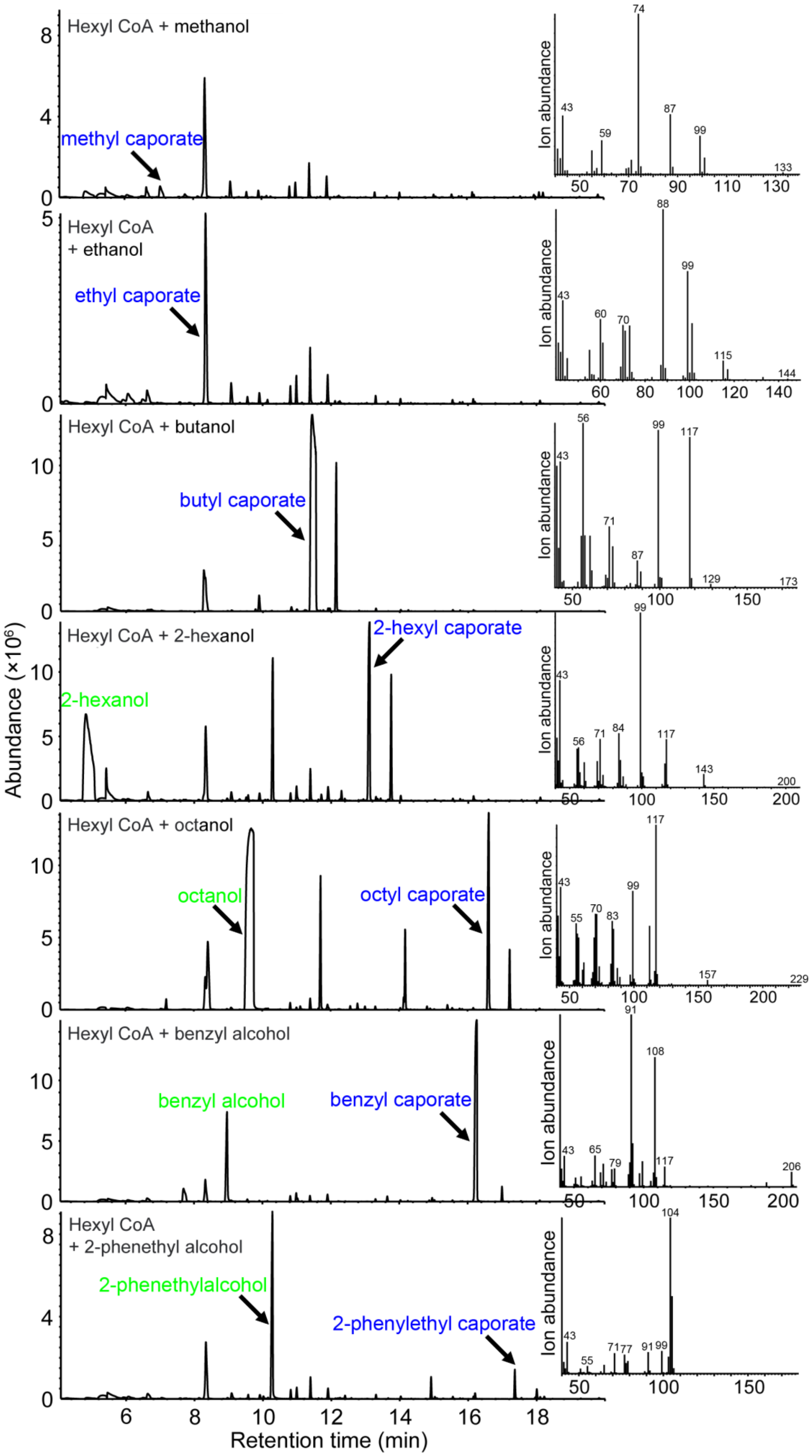

Hexyl CoA + methanol
methyl caporate
Hexyl CoA + ethanol
ethyl caporate
Hexyl CoA + butanol
butyl caporate
Hexyl CoA + 2-hexanol
2-hexanol
2-hexyl caporate
Hexyl CoA + octanol
octanol
octyl caporate
Hexyl CoA + benzyl alcohol
benzyl alcohol
benzyl caporate
Hexyl CoA + 2-phenethyl alcohol
2-phenethylalcohol
2-phenylethyl caporate
Abundance (×10^6)
Retention time (min)
Ion abundance

Supplementary Figure 5. GC-MS analysis of products generated by recombinant LoAAT1 incubated with hexyl-CoA and different alcohol substrates. The alcohol substrates are shown in green while the hexylated products whose mass spectra are displayed as insets are shown in blue.

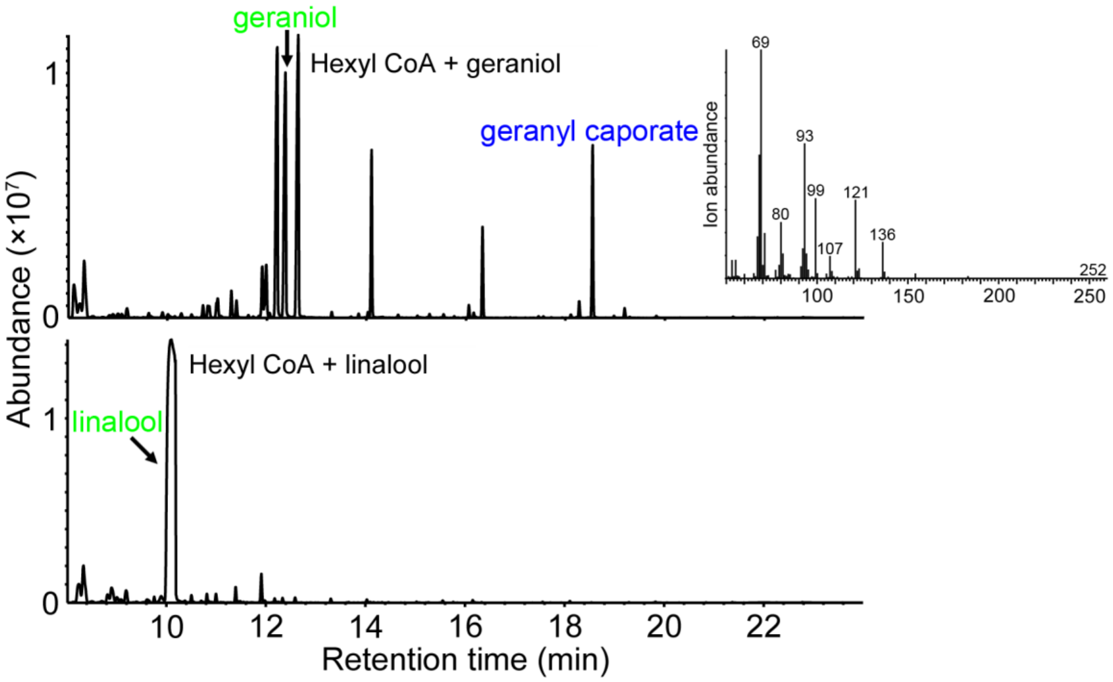

Supplementary Figure 6. GC-MS analysis of products generated by recombinant LoAAT1 incubated with hexyl-CoA and the monoterpene alcohol substrates. The alcohol substrates are shown in green while the hexylated product whose mass spectrum is displayed as an inset are shown in blue.

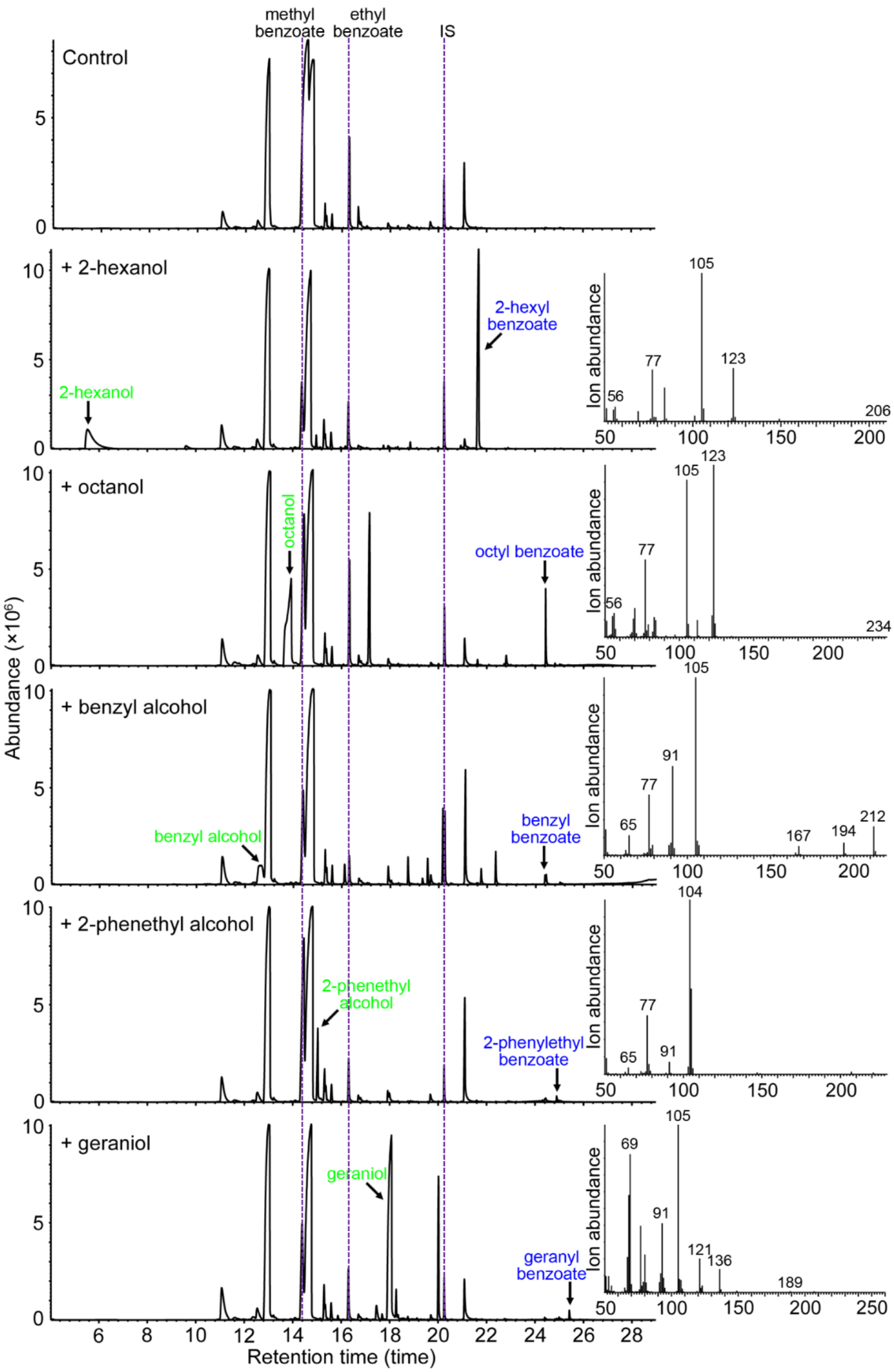

Supplementary Figure 7. Total ion chromatogram of volatiles after infiltration of different alcohol substrates into inner tepals of *Lilium* 'Siberia'. The chromatographic peaks of infiltrated alcohol (green font) and newly formed benzoylated products (blue font) are marked by arrows. The mass spectra of the benzoylated products are displayed as insets. IS: internal standard.

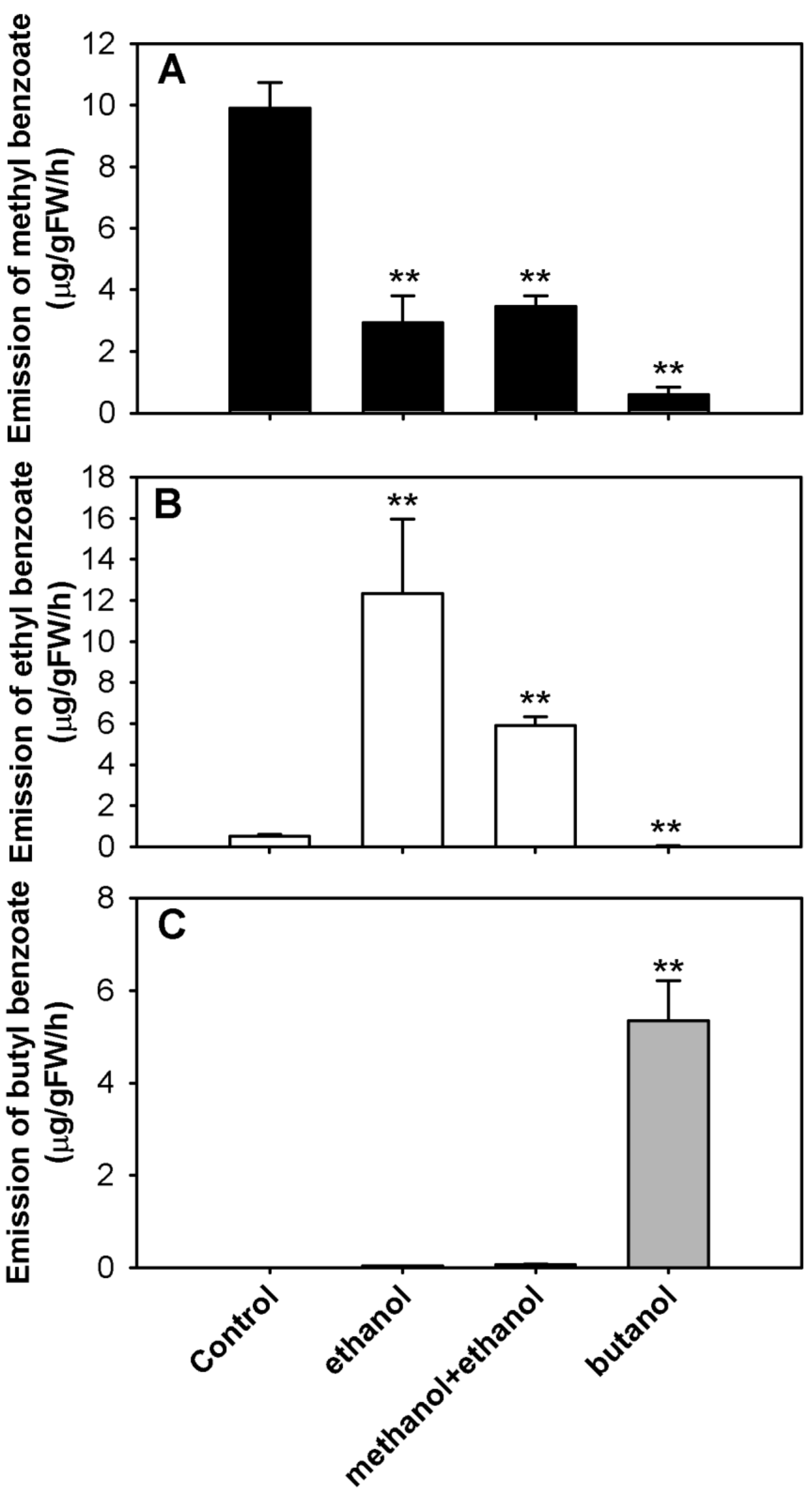

Supplementary Figure 8. Emission of methyl benzoate **(A)**, ethyl benzoate **(B)** and butyl benzoate **(C)** after infiltration of different alcohol substrates into inner tepals of *Lilium* 'Siberia'. Error bars indicate the standard deviation of three biological replicates. Asterisks represent significant differences between treatments and control by Student's *t* test ($^{**}P < 0.01$).

Supplementary Table 1. Summary of the *Lilium* 'Siberia' transcriptome.

Total number of raw reads	20,621,404
Total base pairs (Gbp)	4.17
GC content (%)	50.35
Q20 (%)	92.42
Total number of unigenes	29,837
Mean length of unigenes (bp)	831
N50 (bp)	1,269

Supplementary Table 2. Summary of DGE sequencing during tepal development.

**Description**	**Bud**	**BM_L**	**BM_D**
Total raw reads	12,023,078	8,973,373	8,824,284
Total bases (G)	1.21	0.91	0.89
Q20 (%)	94.35	93.29	93.70
GC content (%)	52.15	50.67	49.97
Total mapped reads	9,007,663	7,609,257	7,420,112
Total mapped reads /Total clean reads (%)	74.92	84.80	84.01

BM_L and BM_D represent the tepals in the blooming stage at the daytime (16:00) and dark (4:00 AM), respectively. DGE: digital gene expression profiling.

Supplementary Table 3. Analysis of putative genes related to benzenoid biosynthesis.

Name	Gene ID	Description	Full -length	Amino acid	Homolog	Identity (%)	Expression quantity (RPKM)		
							Bud	BM_L	BM_D
LoPAL1	Unigene.26868	Phenylalanine ammonialyase	2388	701	AtPAL1 (NP_181241)	78.61	0.33	834.83	391.09
LoPAL2	Unigene.29838	Phenylalanine ammonialyase	2379	713	AtPAL1 (NP_181241)	81.07	365.52	2194.72	1010.22
LoPAL3	Unigene.24577	Phenylalanine ammonialyase	2272	708	AtPAL1 (NP_181241)	79.60	66.92	9.44	0.52
LoPAL4	Unigene.26217	Phenylalanine ammonialyase	2491	757	AtPAL1 (NP_181241)	60.72	146.21	40.69	57.92
LoCNL	Unigene.28102	Cinnamoyl-CoA ligase	2138	525	PhCNL (AEO52693)	61.99	1.77	2165.45	380.15
LoCHD1	Unigene.29358	Cinnamoyl-CoA hydratase/dehydrogenase	2761	724	PhCHD (AFS41246)	72.68	337.20	591.76	968.25
LoCHD2	Unigene.3543	Cinnamoyl-CoA hydratase/dehydrogenase	2689	727	AtMFP2 (At3g06860)	74.72	71.57	90.03	297.79
LoKAT	Unigene.18419	3-Ketoacyl CoA thiolase	1861	459	PhKAT1 (ACV70032)	76.19	897.25	3396.14	4025.46
LoBALD	Unigene.23108	Benzaldehyde dehydrogenase	1930	531	AmBALD (ACM8973)	79.48	30.84	100.72	172.64
LoSABATH1	Unigene.7620	SABATH methyltransferases	1367	356	OsBSMT (XP_467504)	40.69	0.00	2.27	0.56
LoSABATH2	Unigene.29839	SABATH methyltransferases	1490	359	OsBSMT (XP_467504)	39.36	0.82	0.00	0.00
**LoAAT1**	**Unigene.19741**	**Alcohol acyl transferase**	**1905**	**459**	CbBEBT (AAN09796)	**67.57**	**2.68**	**14339.57**	**5341.48**
LoAAT2	Unigene.21917	Alcohol acyl transferase	1495	447	CbBEBT (AAN09796)	52.26	4.38	30.33	18.75
LoAAT3	Unigene.22042	Alcohol acyl transferase	1427	404	CbBEBT (AAN09796)	49.61	0.70	0.00	0.09
LoPME	Unigene.28525	Pectin methylesterase	1754	487	AtPME6 (At1g23200)	54.56	0	3288.07	636.81

BM_L and BM_D represent the tepal in the blooming stage at the daytime (16:00) and dark (4:00 AM), respectively. RPKM: reads per kilobase of exon model per million mapped reads.

Supplementary Table 4. Primer sequences used in this study.

Gene	Forward/reverse primer sequence (5'-3')	Purpose
LoAAT1	ATGGCATCATCCCTCACTTTCT<br>CTATAATTCCCTTTTACCCTTCG	gene isolation
LoAAT1	*Eco*RI-GAATTC ATGGCATCATCCCTCACTTTCT<br>*Hind*III-AAGCTTCGAGGGCAGAAGCAATAAACTGC	bacterial expression
LoAAT1	ATTGTGGTGCCAGTTTGCTTGC<br>CCCTTTTACCCTTCGTTGAACTTC	real-time PCR
LoGAPDH	CACTGCTACTCAGAAAAC<br>ATCAACAACAGACACATC	real-time PCR
